# The non-monotonic dose-effect of resveratrol on testicular steroidogenesis in male arctic foxes (Vulpes lagopus): Mechanisms revealed by integrated transcriptomic and metabolomic analyses

**DOI:** 10.1016/j.vas.2026.100666

**Published:** 2026-04-20

**Authors:** MA Limin, Duan Fuwen, Liu Zijian, Tian Yu, Wu Min, Wei Gongqing

**Affiliations:** a Jilin Agricultural University College of Animal Science and Technology, Changchun, 130118, China; b Key Laboratory of Animal Production and Product Quality & Safety, Ministry of Education, Changchun, 130118, China

**Keywords:** Resveratrol, Arctic fox, Metabolomics, Transcriptomics, Testis, Testosterone

## Abstract

The Arctic fox is a mammal that is intensively farmed for its valuable fur, and its farming industry has developed on a large scale worldwide. However, the current farming of arctic foxes still faces challenges such as reproductive efficiency, which hinder the sustainable development of the industry. Resveratrol is a natural polyphenolic compound found in plants such as grapes and Japanese knotweed. It has attracted significant attention due to its potent antioxidant and anti-inflammatory properties and has been shown to have reproductive protective effects in mammals by regulating the steroid hormone synthesis pathway. In this experiment, 40 foxes were divided into four groups and fed diets containing 0, 10, 50, or 100 mg/kg of resveratrol for two months. The results showed that serum testosterone and luteinizing hormone levels exhibited a non-monotonic dose-response relationship, peaking in the 50 mg/kg group. Integrated transcriptomic and metabolomic analyses indicated that low doses may have no significant effect due to insufficient pathway activation, medium doses likely promote testosterone synthesis by upregulating key steroidogenic genes such as *HSD3B* and *CYP11A1*, while high doses may have limited efficacy due to feedback inhibition and accelerated metabolism. Conclusion: A moderate dose of resveratrol can most effectively enhance testosterone synthesis by modulating the steroidogenic network, providing a basis for its use as a feed additive in reproductive management.

## Highlights

• Optimal resveratrol dose (50 mg/kg) enhances testosterone in Arctic foxes.
• Non-monotonic dose-effect linked to key steroidogenic gene activation.
• Multi-omics reveals interplay between synthesis promotion and metabolic clearance.
• Resveratrol hierarchically upregulates *CYP11A1, HSD3B*, and *CYP17A1* genes.
• High-dose risks: estrogenic feedback and elevated corticoid precursor.

## 1 Introduction

The Arctic fox (Vulpes lagopus), as a valuable fur-bearing animal, occupies an important position in the domestic fur industry due to its dense underfur, elegant coloration, and excellent thermal insulation properties (Liu et al., 2025). The healthy development of its farming industry is of great significance for meeting market demands and promoting the growth of specialized economies. However, the ultimate economic benefits of farming Arctic foxes depend not only on fur quality but also closely on the reproductive efficiency of the population. In the reproductive process, the reproductive capacity of male foxes plays a decisive role. Studies have shown that the age of male foxes is closely related to their reproductive performance, with 2-year-old males typically exhibiting optimal mating performance (Wang & Hua, 2007). The reproductive capacity of male foxes is fundamentally determined by the developmental status and physiological functions of their testes (Sun et al., 2001). Healthy testes are essential for maintaining normal androgen secretion and efficient spermatogenesis (Xiao et al., 2024), directly influencing semen quality and the breeding value of male foxes. However, current specialized research on the testicular biology and nutritional regulation mechanisms of Arctic foxes remains limited. Existing literature predominantly focuses on the reproductive management of female foxes, resulting in a lack of theoretical support for precise nutritional strategies tailored to male foxes.

Resveratrol, a natural polyphenolic compound, has demonstrated beneficial effects as a feed additive in improving animal growth performance, antioxidant status, and reproductive potential (Wei and Zuo, 2025; Xue et al., 2025; Zheng, 2025). For instance, studies on boars have shown that dietary resveratrol supplementation significantly increases serum levels of follicle-stimulating hormone (FSH), luteinizing hormone (LH), and testosterone, effectively enhancing semen volume, sperm density, and motility, while reducing oxidative stress and inflammatory markers (Guo et al., 2024). Its mechanism of action is closely related to the regulation of testicular proteome expression and the mitigation of oxidative damage. Furthermore, research has found that resveratrol can effectively alleviate heat stress-induced testicular functional impairment in boars and improve their summer semen quality by inhibiting the AGEs-RAGE signaling pathway in testicular Leydig cells (Peng et al., 2025). In small animal models, dietary resveratrol supplementation in mice increased testicular index and semen quality, improved testicular oxidative damage, reduced mitochondrial damage and acrosomal abnormalities (Li, 2021; Luo, 2020; Meydanli et al., 2018; Zhang, 2018). It also enhanced testicular testosterone secretion and mitigated abnormal spermatogenesis by suppressing the decline in autophagy levels and mitochondrial function in aged Leydig cells (Abdelali et al., 2016; Lin, 2021). Additionally, this compound has shown clear therapeutic effects against chemotoxicant-induced spermatogenic dysfunction in rats, with mechanisms involving the inhibition of germ cell apoptosis and the promotion of seminiferous epithelium repair (Jiang et al., 2007). A review published in 2022 systematically summarized the beneficial effects of resveratrol on the mammalian male reproductive system and explicitly pointed out its development prospects as a novel functional feed additive (Shao et al., 2022). However, a key academic controversy exists in current research: the effects of resveratrol are not always simply positively correlated with dose. In rat models of cerebral ischemia-reperfusion injury and mouse models of coronary atherosclerosis, resveratrol improved neurological function, reduced cerebral infarct volume, lowered blood lipids, and exerted anti-atherosclerotic effects in a dose-dependent positive manner (Zhang et al., 2017; Zhu et al., 2019). It should be noted that the dose ranges examined in these two studies were all within the safe low-dose range of resveratrol. The highest dose used by Zhu et al., 150 mg/kg·d⁻¹, is far below the known no-observed-adverse-effect level of 200 mg/kg/d (Johnson et al., 2011), and did not reach higher doses that might cause toxicity, metabolic saturation, or a plateau phase of effect. Therefore, whether the observed "positive correlation" can be extrapolated to higher dose ranges remains unclear. In contrast, for laying hens, a medium dose of resveratrol was most effective in improving laying performance, egg quality, and reproductive hormone levels (Ju et al., 2025); similarly, in finishing pigs, the medium-dose group showed the most significant improvements in growth performance, antioxidant function, and economic benefits (Liu, 2025). Although the above two studies identified non-monotonic dose-response relationships (medium dose optimal), both were limited to the description of phenotypic indicators and did not conduct in-depth molecular analyses to explain the underlying reasons for this phenomenon. In terms of lipid metabolism regulation, Song et al. (2023) reported on one hand that dietary supplementation with 600 mg/kg resveratrol promoted intramuscular fat deposition in goats via the PI3K-AKT pathway, and on the other hand found in vitro that 40 μmol/L resveratrol inhibited the proliferation and adipogenesis of mouse 3T3-F442A preadipocytes. Notably, both parts of this experiment examined only a single dose level (only 600 mg/kg in vivo in goats, and only 40 μmol/L in vitro), with no dose gradient set. Therefore, this study can only demonstrate that different doses of resveratrol produce different effects in different systems, but cannot draw any conclusion about dose-response relationships or "bidirectional regulation." The contradictory effects described above are closely related to differences in dose, species, and target tissues, highlighting the complexity of resveratrol's mechanism of action and the deficiencies in existing evidence regarding dose systematization and mechanistic depth.

Based on the aforementioned background, this study aims to systematically investigate the effects of dietary resveratrol supplementation on testicular function in male Arctic foxes through a feeding trial, with a specific focus on elucidating the underlying molecular mechanisms from the perspective of testosterone synthesis regulation. This study establishes two core objectives: First, to clarify the dose-effect relationship of resveratrol in male Arctic foxes by setting low, medium, and high concentration gradients, thereby determining the optimal dosage for improving reproductive performance. Second, to utilize multi-omics technologies to deeply explore the mechanism by which resveratrol influences the testosterone synthesis pathway. To this end, the study will integrate transcriptomics and metabolomics analyses. This approach is expected to systematically reveal the molecular network through which resveratrol regulates testosterone synthesis in male Arctic foxes, providing a solid theoretical foundation for its scientific application in the efficient farming of this species.

## 2 Materials and methods

### 2.1 Animals and experimental design

A single‑factor design with multiple dose levels was used to evaluate the effects of resveratrol on testicular function in Arctic foxes. Forty healthy, seven‑month‑old male foxes with uniform body size and no evident abnormalities (e.g., unilateral cryptorchidism) were obtained from Harbin Hualong Feed Development Co., Ltd. (Harbin, China). The animals were randomly divided into four groups of ten each: a control group (GDZZ, 0 mg resveratrol/kg diet), a low‑dose group (GM1, 10 mg/kg), a medium‑dose group (GM2, 50 mg/kg), and a high‑dose group (GM3, 100 mg/kg). Resveratrol (purity ≥ 99%) was supplied by Xi’an Zebang Biotechnology Co., Ltd. (Xi’an, China).

All foxes were individually housed in outdoor cages at a fur‑animal breeding base. They were fed once daily at 9:00 AM, with the feeding process completed within approximately two hours; no personnel approached the cages at other times. After a ten‑day acclimation period, resveratrol supplementation began. The compound was separately administered into each animal’s feeding trough together with the basal diet to ensure accurate dose delivery. Any fox showing abnormal signs during the trial was immediately isolated and managed separately.

### 2.2 Sample collection and processing

Upon reaching sexual maturity, the foxes were transported from the rearing area to the slaughter area. Blood samples (approximately 10 mL in total) were collected via the hind limb venous method using coagulation-promoting tubes. After immediate centrifugation, serum samples were temporarily stored on dry ice. The foxes were then humanely euthanized by electrical stunning (Korhonen et al., 2009), and both testes were collected and preserved on dry ice. All samples were subsequently transferred to a –80 °C freezer for long-term storage. Six foxes from each group were sampled for analysis.

### 2.3 Hormone assay

Serum samples stored at –80 °C were thawed at 4 °C and analyzed using enzyme-linked immunosorbent assay (ELISA) kits for testosterone (T), follicle-stimulating hormone (FSH), and luteinizing hormone (LH) (Shanghai Langdun Biotechnology Co., Ltd., China). All assays were performed strictly according to the manufacturer’s instructions. Absorbance was measured using a microplate reader, and hormone concentrations were calculated based on a logistic curve fitted from standard calibrators.

### 2.4 Transcriptomic sequencing and analysis

Total RNA was extracted from testicular tissue using Trizol reagent (Invitrogen Life Technologies). RNA concentration, purity, and integrity were assessed with a NanoDrop spectrophotometer (Thermo Scientific). Sequencing libraries were constructed from 3 μg of total RNA per sample. Paired-end sequencing was performed on an Illumina NovaSeq 6000 platform.

Raw sequencing data were filtered to obtain clean reads, which were then aligned to the Arctic fox reference genome. Gene expression levels were quantified based on the alignment results. Downstream analyses, including differential expression analysis, functional enrichment analysis, and gene set enrichment analysis (GSEA), were performed on the Paisenor Gene Cloud Platform.

### 2.5 Untargeted metabolomic profiling

Metabolites were extracted from serum using a liquid-liquid extraction method with an organic solvent system (methanol/acetonitrile/water). Data acquisition was performed on a Thermo Orbitrap Exploris 120 mass spectrometer controlled by Xcalibur software (version 4.7, Thermo Scientific) in both positive and negative ion modes with data-dependent acquisition (DDA).

Raw data were converted to .mzXML format using ProteoWizard and processed with XCMS for peak alignment, retention time correction, and peak area extraction. Metabolite identification and data preprocessing were carried out before quality assessment and statistical analysis.By matching the retention time, mass (with a mass error of <10 ppm), MS/MS fragmentation spectra, collision energy, and other information against a database, the metabolites in biological samples are structurally identified. The identification results are then strictly manually rechecked and confirmed, achieving an identification level of Level 2 or higher.

### 2.6 Data integration and statistical analysis

Hormone data were analyzed using ELISAcalc with a logistic regression model. Transcriptomic and metabolomic datasets were analyzed and visualized on the Paisenor Gene Cloud Platform, which included differential analysis, functional enrichment, GSEA, and integrative multi-omics correlation analysis. Statistical significance was set at *p* < 0.05, unless otherwise specified.

## 3 Results

### 3.1 Levels of luteinizing hormone (LH), testosterone (T), and follicle-stimulating hormone (FSH)

**Fig. 1:**
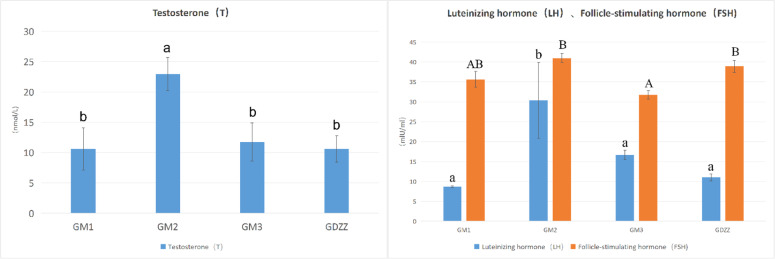
Hormone levels of the experimental group and the control group. GDZZ, control group (0 mg/kg resveratrol); GM1, low-dose group (10 mg/kg resveratrol); GM2, medium-dose group (50 mg/kg resveratrol); GM3, high-dose group (100 mg/kg resveratrol).The blue bars represent luteinizing hormone (LH, in mIU/mL), the red bars represent testosterone (T, in nmol/L), and the green bars represent follicle-stimulating hormone (FSH, in mIU/mL).Data are presented as mean ± SEM. Different lowercase letters indicate significant differences (*P* < 0.05), different uppercase letters indicate highly significant differences (*P* < 0.01), and the same letter indicates no significant difference (*P* > 0.05).

The changes in Luteinizing Hormone (LH) and Testosterone levels followed highly consistent trends: hormone levels in the medium-dose group were significantly higher than those in the low-dose, high-dose, and control groups (Fig. 1).

### 3.2 Transcriptomic results

All testicular tissue samples were sequenced on the Illumina platform. The original image files were converted by the platform's built-in software into raw data in FASTQ format. As shown in Table 1, all samples demonstrated excellent data quality metrics. Specifically, the average Q20 and Q30 values exceeded 99% and 97%, respectively, while both Clean Reads (%) and Clean Data (%) were above 99.7%. These results indicate that the sequencing data are highly reliable and accurate, fully meeting the stringent requirements for subsequent bioinformatics analysis.

**Table 1:** Sequencing data quality assessment table.

**Sample**	**GM1**	**GM2**	**GM3**	**GDZZ**
**Raw Reads No**	49974662 ± 2862015	57242761 ± 4331501	46517385 ± 4029000	47280633 ± 3697000
**Clean Reads No**	49956492 ± 2862133	57218286 ± 4330127	46499313 ± 4023000	47259624 ± 3686000
**Raw Bases (bp)**	7496199300 ± 429300000	8586414200 ± 649725000	6977607800 ± 603800000	7092094900 ± 553600000
**Clean Data (bp)**	7479165912 ± 428200000	8566234900 ± 648700000	6960110300 ± 601800000	7073133371 ± 552000000
**Q30 (bp)**	7309399672 ± 410400000	8408549906 ± 636300000	6828519234 ± 584400000	6942702728 ± 542600000
**GC (%)**	47.86 ± 0.16	48.0033 ± 0.0067	47.83 ± 0.11	48.34 ± 0.13
**Q20 (%)**	99.310 ± 0.029	99.4267 ± 0.0033	99.413 ± 0.027	99.4133 ± 0.0088
**Q30 (%)**	97.52 ± 0.11	97.93 ± 0.01	97.88 ± 0.10	97.890 ± 0.032
**Clean Reads (%)**	99.9633 ± 0.0033	99.96 ± 0.0033	99.9633 ± 0.0033	99.9567 ± 0.0033
**Clean Data (%)**	99.7733 ± 0.0033	99.7633 ± 0.0088	99.7533 ± 0.0088	99.7333 ± 0.0033

Table notes: Sample: Sample name; Raw_Read_No: Total number of reads; Clean Read No: Number of high-quality sequence reads; Raw_Bases(bp): Total number of bases; Clean Data(bp): Number of high-quality sequence bases; Q30(bp): Total number of bases with a base recognition accuracy ≥ 99.9%; GC(%): GC content; Q20(%): Percentage of bases with a base recognition accuracy ≥ 99%; Q30(%): Percentage of bases with a base recognition accuracy ≥ 99.9%; Clean Reads(%): Percentage of high-quality sequence reads relative to total sequencing reads; Clean Data(%): Percentage of high-quality sequence bases relative to total sequencing bases.

**Fig. 2:**
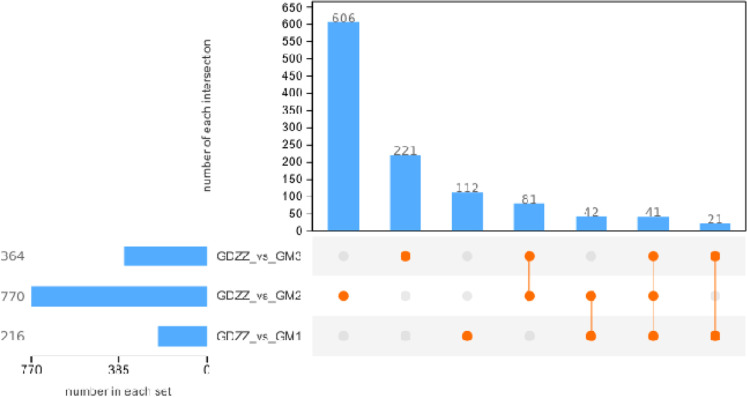
Differentially expressed genes UpSet diagram.Each vertical bar in the top panel (number in each set) indicates the total number of differential genes identified in a single comparison (treatment vs. control). The connected dots in the bottom matrix represent intersections among multiple comparisons, and the corresponding vertical bar (number of each intersection) shows the count of shared differential genes across those groups. A single dot in the matrix indicates the unique differential genes for that group, while a line connecting multiple dots represents the common differential genes across the connected groups. Differential genes were screened with thresholds of *p* < 0.05 and |log₂(fold change)| > 1. Groups are: low‑dose (GM1, 10 mg/kg resveratrol), middle‑dose (GM2, 50 mg/kg resveratrol), high‑dose (GM3, 100 mg/kg resveratrol), and control (GDZZ, 0 mg/kg resveratrol).

The results of differential expression analysis of testicular transcriptomic data are shown in Fig. 2. Compared to the control group (GDZZ), the low-dose (GM1), medium-dose (GM2), and high-dose (GM3) groups showed 216, 770, and 364 differentially expressed genes (DEGs), respectively. Notably, the number of DEGs did not increase monotonically with dosage but instead peaked at the medium dose and then decreased significantly at the high dose.

### 3.3 Metabolomics results

**Fig. 3:**
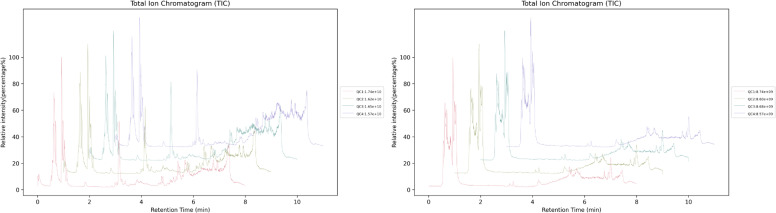
Overlay of total ion chromatograms (TICs) from quality control (QC) and representative experimental samples.The horizontal axis represents the retention time of each chromatographic peak, and the vertical axis represents the peak intensity.

The stability of the LC-MS/MS analysis was confirmed by overlaying the total ion chromatograms (TICs) of all quality control (QC) samples. As shown in Fig. 3, The close overlap of chromatographic peaks across all samples demonstrates stable instrument performance and high reproducibility of metabolite extraction and analysis throughout the experiment.

**Fig. 4:**
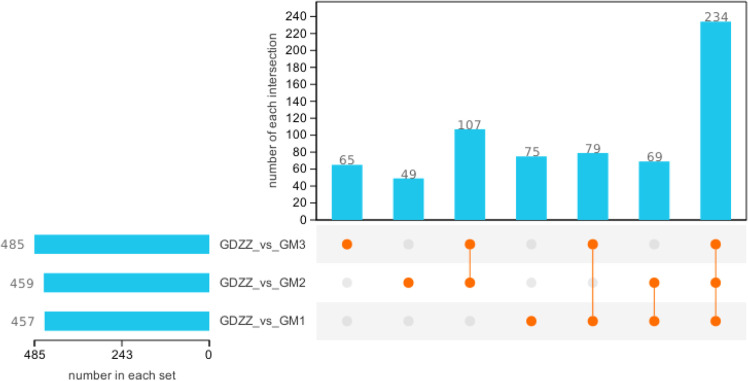
Differential metabolites UpSet diagram.Each vertical bar in the top panel (number in each set) indicates the total number of differential metabolites identified in a single comparison (treatment vs. control). The connected dots in the bottom matrix represent intersections among multiple comparisons, and the corresponding vertical bar (number of each intersection) shows the count of shared differential metabolites across those groups. A single dot in the matrix indicates the unique differential metabolites for that group, while a line connecting multiple dots represents the common differential metabolites across the connected groups. Differential metabolites were screened with thresholds of *p* < 0.05 and fold change > 1. Groups are: low‑dose (GM1, 10 mg/kg resveratrol), middle‑dose (GM2, 50 mg/kg resveratrol), high‑dose (GM3, 100 mg/kg resveratrol), and control (GDZZ, 0 mg/kg resveratrol).

Untargeted metabolomics analysis of testicular tissues demonstrated that resveratrol at different doses significantly altered the metabolic profiles (Fig. 4). Compared with the control group (GDZZ), the low- (GM1), medium- (GM2), and high-dose (GM3) groups exhibited 457, 459, and 485 differentially abundant metabolites, respectively. The total number of differential metabolites showed an increasing trend with higher doses.

### 3.4 Integrated analysis of transcriptomic and metabolomic data between GDZZ and GM2

**Fig. 5:**
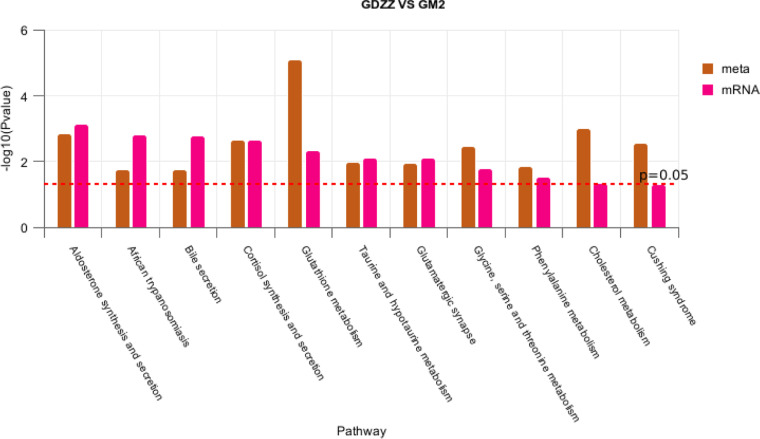
Co-enrichment pathway map of GDZZ VS GM2.The horizontal axis represents the names of the metabolic pathways shared by the transcriptome and metabolome, and the vertical axis represents the *p*-log10 (*p*-value) values of the enrichment analysis of the two omics. The brown bars represent the metabolome, the red bars represent the transcriptome, and the red dotted line indicates the position of p=0.05. The results above the dotted line are those with *p* values less than 0.05.

A KEGG pathway co-enrichment analysis was performed on the GDZZ and GM2 groups (Fig. 5). 11 key pathways were identified, whose functions can be primarily categorized into four aspects: steroid hormone synthesis (Aldosterone synthesis and secretion, Cortisol synthesis and secretion, Cholesterol metabolism, and Cushing syndrome), immune privilege (African trypanosomiasis and Bile secretion), antioxidant stress response (Glutathione metabolism, Taurine and hypotaurine metabolism, and Glycine, serine and threonine metabolism), and neuroendocrine regulation (Glutamatergic synapse and Phenylalanine metabolism).

### 3.5 Key Pathways and genes in testosterone synthesis and metabolism

**Fig. 6:**
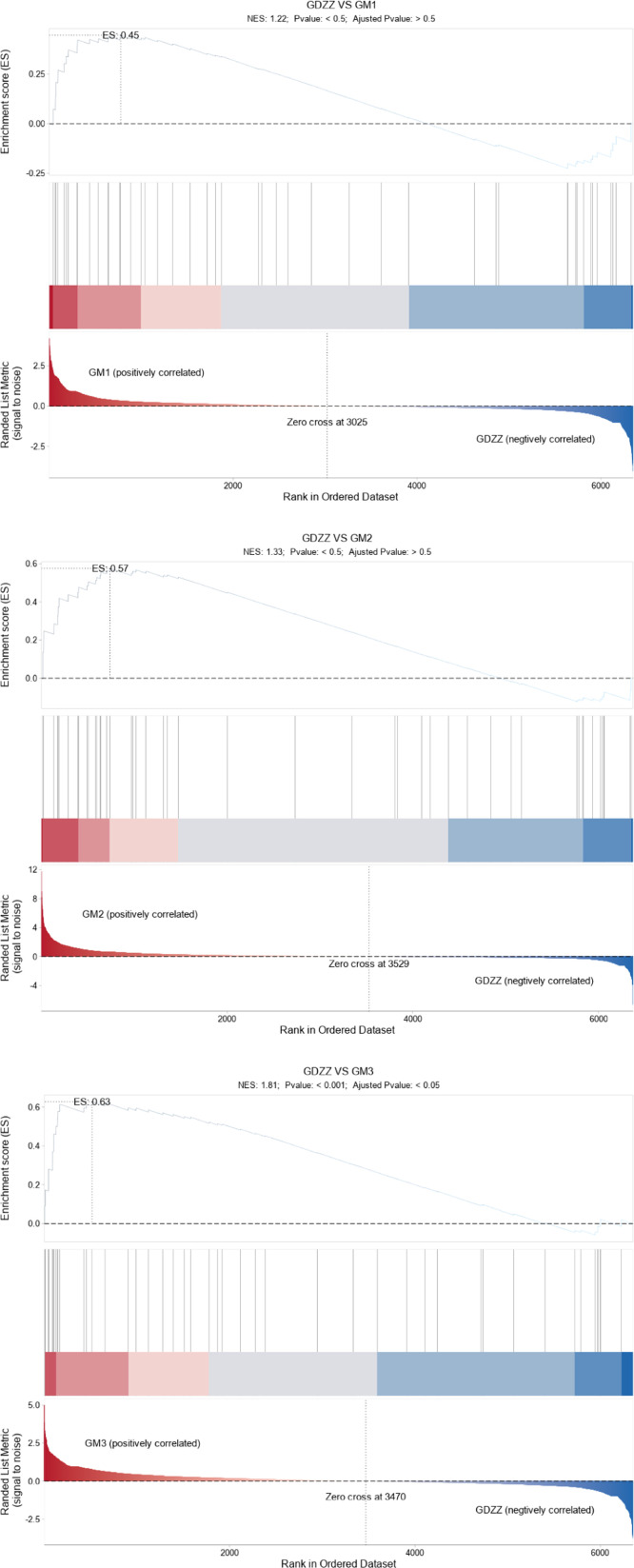
GSEA enrichment plot for the steroid hormone biosynthesis pathway in testes.The GSEA plot consists of three panels: (Top) The distribution curve of the enrichment score (ES), where the peak (farthest from the zero point) indicates the ES value for the gene set; (Middle) Vertical lines mark the positions of genes from the set within the ranked list, with each line representing an individual gene; (Bottom) The overall ranked distribution of all genes, with red and blue areas indicating genes upregulated in the treatment and control groups, respectively.

Correlation analysis indicated alterations in local steroid metabolism pathways in testicular tissue, accompanied by a significant increase in testosterone levels. To explore the potential underlying mechanisms at the systemic level, we performed gene set enrichment analysis (GSEA) on the KEGG pathway "steroid hormone biosynthesis," which encompasses the complete steroid hormone synthesis process. The results revealed that this pathway exhibited significant positive enrichment across all resveratrol treatment groups, with enrichment scores increasing progressively in response to higher treatment concentrations (Fig. 6). Further analysis identified core genes driving the enrichment of this pathway (Table 2), including: *CYP11A1*, the rate-limiting enzyme in steroid synthesis responsible for catalyzing the conversion of cholesterol to pregnenolone; key enzymes in the testosterone synthesis pathway, *HSD3B* and *CYP17A1*; and key enzymes involved in testosterone glucuronidation metabolism, *UGT2A3* and *UGT2B31. CYP11A1* and *UGT2A3* were significantly upregulated in both the GM2 and GM3 groups and were identified as core-enriched genes. *HSD3B* showed significant upregulation and core enrichment across all experimental groups. *CYP17A1* exhibited significant upregulation and core enrichment only in the GM3 group.Although the expression changes of UGT2B31 and CYP19A1 were not statistically significant among all groups, UGT2B31 was consistently identified as a core enrichment gene, while CYP19A1 showed a downward trend.

**Table 2:** Enrichment and Expression details of core genes from GSEA in the Testosterone synthesis and metabolism pathway.

**Gene Symbol**	**Treatment**	**log₂FC**	***P*-value**	**Regulation**	**RANK METRIC SCORE**	**Running ES**	**Core Enrichment**
***CYP11A1***	GM1	0.29	*P* < 0.001	ns	0.2927	0.4397	No
	GM2	1.43	*P* < 0.001	up	1.4285	0.4380	Yes
	GM3	2.08	*P* < 0.001	up	2.0803	0.2251	Yes
***HSD3B***	GM1	1.18	*P* < 0.001	up	1.1757	0.3036	Yes
	GM2	1.85	*P* < 0.001	up	1.8497	0.4186	Yes
	GM3	3.34	*P* < 0.001	up	3.3360	0.0908	Yes
***CYP17A1***	GM1	-0.02	0.4505	ns	-0.0199	0.1290	No
	GM2	0.91	*P* < 0.001	ns	0.9069	0.4843	Yes
	GM3	1.56	*P* < 0.001	up	1.5610	0.5013	Yes
***UGT2A3***	GM1	0.04	0.9547	ns	0.0371	0.2332	No
	GM2	4.57	*P* < 0.001	up	4.5660	0.2476	Yes
	GM3	1.70	*P* < 0.001	up	1.7017	0.4168	Yes
***UGT2B31***	GM1	1.05	0.4083	ns	1.0512	0.3400	Yes
	GM2	1.12	0.3528	ns	1.1213	0.4765	Yes
	GM3	1.76	0.1172	ns	1.7572	0.3715	Yes
***CYP19A1***	GM1	-0.16	0.1535	ns	-0.1588	-0.1081	No
	GM2	-0.55	*P* < 0.001	ns	-0.5517	-0.0977	No
	GM3	-0.64	*P* < 0.001	ns	-0.6360	-0.0289	No

Notes: GM1: low-dose group (10 mg/kg resveratrol); GM2: medium-dose group (50 mg/kg resveratrol); GM3: high-dose group (100 mg/kg resveratrol); Treatment: experimental treatment groups; log₂FC: log₂-transformed fold change; P-value: significance probability; Regulation: expression regulation trend, Genes with |log₂FC| ≥ 1 and P < 0.01 were defined as differentially expressed genes,“up” for significant up-regulation and “ns” for no significant difference; RANK METRIC SCORE: rank metric score; Running ES: running enrichment score; Core Enrichment: whether a gene is identified as a core enrichment gene (“Yes” or “No”).

### 3.6 Key Metabolites in testosterone synthesis and metabolism

**Fig. 7:**
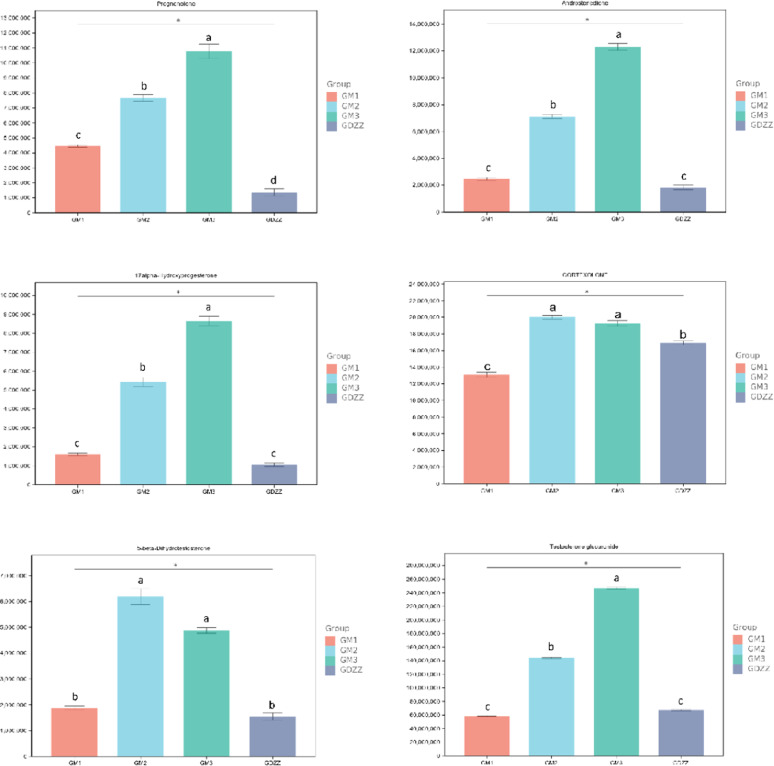
Bar chart of the content of testosterone related metabolites.Based on untargeted metabolomics data from testicular tissues, it displays the relative abundance of key substances in the testosterone synthesis pathway across different treatment groups.GDZZ, control group (0 mg/kg resveratrol); GM1, low-dose group (10 mg/kg resveratrol); GM2, medium-dose group (50 mg/kg resveratrol); GM3, high-dose group (100 mg/kg resveratrol).Data are presented as mean ± SEM. Different lowercase letters indicate significant differences (*P* < 0.05), different uppercase letters indicate highly significant differences (*P* < 0.01), and the same letter indicates no significant difference (*P* > 0.05).

The metabolomics results are shown in Fig. 7. The levels of testosterone upstream precursors—pregnenolone, 17α-Hydroxypregnenolone, and androstenedione—all demonstrated a progressive increase from the control group to the high-dose group, reaching peak levels in the high-dose group. The level of the testosterone downstream metabolite, 5β-dihydrotestosterone, was significantly higher in the medium- and high-dose groups compared to the control group, but its content in the high-dose group was significantly lower than that in the medium-dose group. Testosterone glucuronide showed an increasing trend from the control group to the high-dose group, peaking in the high-dose group.Cortodoxone levels were highest in the medium-dose group, slightly lower in the high-dose group but still elevated compared to the control group.Furthermore, while a significant increase in estrone levels was observed exclusively in GM2, an overall rising trend was evident across the three dose groups.

## 4 Discussion

This study presents the first integrated transcriptomic and metabolomic analysis of the effects of varying concentrations of dietary resveratrol on testosterone production in male Arctic foxes (Vulpes lagopus). The results reveal a non-monotonic dose-response relationship, where serum testosterone (T) levels peaked in the medium-dose group (GM2; 50 mg/kg), while both the low-dose (GM1; 10 mg/kg) and high-dose (GM3; 100 mg/kg) groups yielded suboptimal outcomes.

Testosterone biosynthesis proceeds via two primary pathways. The Δ^5^ pathway proceeds as follows: Cholesterol − (*CYP11A1*) → Pregnenolone(Δ⁵) − (*CYP17A1*) → 17α-Hydroxypregnenolone(Δ⁵) − (*CYP17A1*) → Dehydroepiandrosterone (DHEA,Δ⁵) − (*HSD3B*) → Androstenedione(Δ⁴) − (*HSD17B3*) → Testosterone(Δ⁴) . The Δ^4^ pathway proceeds as follows: Cholesterol −(*CYP11A1*) → Pregnenolone(Δ⁵) − (*HSD3B*) → Progesterone(Δ⁴) − (*CYP17A1*) → 17α-Hydroxyprogesterone(Δ⁴) − (*CYP17A1*) → Androstenedione(Δ⁴) − (*HSD17B3*) → Testosterone(Δ⁴) (Conley & Bird, 1997; Hall et al., 1969; Zirkin & Papadopoulos, 2018).

Low-dose group (GM1; 10 mg/kg): Testosterone (T) levels showed no significant difference compared with the control group (GDZZ; 0 mg/kg). Transcriptomic analysis revealed that low-dose treatment selectively upregulated *HSD3B*, a gene downstream in the steroidogenesis pathway, whereas no significant changes were observed in the expression of two key rate-limiting enzymes located at the initiation and key branch points of the pathway, namely *CYP11A1* and *CYP17A1* (Table 2). In the Δ4 pathway of testosterone synthesis, *CYP11A1* catalyzes the initial conversion of cholesterol to pregnenolone, while *HSD3B* catalyzes the subsequent conversion of pregnenolone to progesterone (Ye et al., 2011). Notably, despite unchanged *CYP11A1* expression, pregnenolone levels were significantly elevated (Fig. 7). Miller et al. (2011), in their review in Endocrine Reviews, discussed the complexity of steroidogenesis, noting that steroid hormones can be synthesized via multiple metabolic pathways – a known feature that correlates with the observed accumulation of pregnenolone. Collectively, these observations are consistent with the possibility that, at the low dose, insufficient substrate supply may act as a limiting factor for overall pathway flux, aligning directionally with the lack of increase in testosterone production. This correlative observation is in agreement with the concept proposed by Payne and Hales that total output of the steroidogenic pathway is limited by the slowest step among multiple key points (Payne & Hales, 2004). It should be noted that this interpretation is based on correlative changes in gene expression, and future studies directly measuring enzyme activities and metabolic flux are required for validation.

Medium-dose group (GM2; 50 mg/kg): This treatment was associated with the most pronounced elevation in testosterone levels, with T reaching its peak (Fig. 1). Transcriptomic analysis showed that, compared with the low dose, the medium dose not only sustained upregulation of *HSD3B* but also significantly upregulated *CYP11A1*, the gene encoding the initial rate-limiting enzyme (Table 2). However, *CYP17A1* expression remained unchanged (Table 2). Previous studies have demonstrated that *CYP17A1* activity plays a critical role in determining whether metabolic flux is directed toward androgen or cortisol synthesis (Lee et al., 2023; Yoshimoto & Auchus, 2015). In the present study, the lack of *CYP17A1* upregulation was associated with elevated levels of 17α-hydroxyprogesterone and corticosterone. According to known metabolic pathways, 17α-hydroxyprogesterone can be converted to corticosterone via *CYP21A2* (Loke et al., 2021), and corticosterone can be further metabolized to cortisol (Knuechel et al., 2024). Our metabolomics analysis revealed a significant increase in corticosterone levels in the medium-dose group. Furthermore, integrated transcriptomic and metabolomic association analysis showed significant enrichment of pathways related to "cortisol synthesis and secretion" and "Cushing syndrome" (Fig. 5). These results are consistent with a scenario in which a portion of steroidogenic metabolic flux may be shunted toward adrenal corticosteroid synthesis. Concurrently, expression of *UGT2A3*, a gene involved in hormone inactivation, was significantly upregulated. Levels of its potential reaction products, 5β-dihydrotestosterone (Swerdloff et al., 2017) and testosterone glucuronide, were also significantly elevated (Fig. 7). The *UGT* family of enzymes facilitates the excretion of steroid hormones by catalyzing their glucuronidation (Chouinard et al., 2006; Dang et al., 2016; Meech et al., 2019). These observations are correlated with an activated state of testosterone metabolic clearance. Taken together, these findings are consistent with the hypothesis that, under medium-dose resveratrol treatment, there may exist a transient "synthetic advantage" – i.e., the synthesis rate relatively exceeds the clearance rate – which directionally aligns with the observed peak in testosterone. The precise regulatory nodes maintaining this dynamic equilibrium require further investigation using tracer-based metabolic flux experiments.

High-dose group (GM3; 100 mg/kg): Transcriptomic analysis revealed upregulation of *CYP11A1, HSD3B*, and *CYP17A1* genes. Despite this global activation, testosterone levels declined from the peak observed in the medium-dose group and were not significantly different from those in the control group (GDZZ; 0 mg/kg) (Fig. 1). Metabolomics analysis indicated that the androstenedione level in the GM3 group was the highest among all treatment groups (Fig. 7). Transcriptomic data showed upregulation of key genes promoting androstenedione synthesis (*CYP11A1, HSD3B*) (Table 2), whereas *HSD17B3* (17β-hydroxysteroid dehydrogenase type 3), responsible for converting androstenedione to testosterone, was not detected to be upregulated (Chen, 2020; Gonçalves et al., 2022; Lawrence et al., 2022). This molecular pattern of "enhanced synthesis but blocked conversion" is consistent with the observed accumulation of androstenedione. An in vitro study on testicular hormone regulation in rhesus monkeys suggested that Sertoli cells can convert androstenedione to estradiol (Wang et al., 2002). Studies by Baddela et al., 2020; Vrtačnik et al., 2014; and Moutard et al., 2023 have indicated that excess androstenedione can be converted to estrogens via aromatization. According to the steroid metabolic pathway, *CYP19A1* catalyzes the conversion of androstenedione to estrone. In this study, *CYP19A1* expression showed a decreasing trend (non-significant) (Table 2), whereas estrone levels showed an increasing trend (non-significant) (Table 3), which may be associated with androstenedione accumulation. Estrone can be further converted to the more active estradiol (Andersson & Moghrabi, 1997). Resveratrol has been reported to possess estrogen-like effects (Qasem, 2020). The combined presence of endogenous estrogens (estrone, estradiol) and exogenous resveratrol is associated with conditions that trigger classical endocrine negative feedback: when estrogenic activity exceeds a certain threshold, it can suppress pituitary LH secretion, returning LH levels to the homeostatic range (Christian et al., 2008; Li et al., 2020; Liu, 2020; Maeda et al., 1996; Oduwole et al., 2021; Shaw et al., 2010). Hormone measurements from this study showed that LH was significantly higher in the medium-dose group than in the control group, whereas in the high-dose group, LH decreased significantly compared with the medium-dose group and returned to control levels, consistent with the expected negative feedback regulation. Meanwhile, the high-dose group exhibited a non-significant increasing trend in estrone levels and a decrease in testosterone levels, which is also associated with testosterone suppression under negative feedback (Wu et al., 2021). Additionally, transcriptomic data showed that *UGT2A3* expression in the high-dose group was lower than that in the medium-dose group but higher than that in the control group, and testosterone glucuronide levels were the highest among all treatment groups (Fig. 7), consistent with a persistently active state of testosterone conjugation and metabolism. In summary, high-dose resveratrol treatment is associated with the following concurrent observations: androstenedione accumulation, lack of *HSD17B3* upregulation, non-significant increase in estrone accompanied by non-significant downregulation of *CYP19A1*, an LH negative-feedback pattern (increase at medium dose followed by return to control levels at high dose), and persistent conjugation metabolism reflected by *UGT2A3* expression and testosterone glucuronide levels. These phenomena collectively align directionally with the observed constrained net testosterone production in the high-dose group. It should be noted that the above correlative observations are based on integrated transcriptomic, metabolomic, and hormone level analyses; the specific causal relationships (e.g., metabolic flux shunting, relative contribution of negative feedback, and true synergy between endogenous and exogenous agents) require further validation through enzyme activity assays, metabolic flux tracing, detection of pituitary LH expression, or receptor blockade experiments.

**Table 3:** Changes in estrone levels in the experimental group.

**Group**	**Mean Control**	**Mean Treat**	**log2FoldChange**	***P* value**	**Regulation**
**GM1**	17026689.82 ± 129425.6	17570029.89 ± 293829.6	0.0453	0.1971	No diff
**GM2**	17026689.82 ± 129425.6	18109353.36 ± 273458.5	0.0889	0.0373	Up
**GM3**	17026689.82 ± 129425.6	18144835.34 ± 412960.5	0.0918	0.0980	No diff

Table notes: meanControl: Mean gene expression value for the control group (0 mg/kg resveratrol). meanTreat: Mean gene expression value for each treatment group (GM1: 10 mg/kg, GM2: 50 mg/kg, GM3: 100 mg/kg resveratrol). log2FoldChange: Log2 fold change value, representing the expression change multiple of the treatment group relative to the control group. *P* value: Significance *p*-value for differential expression. Regulation: Determination of differential expression status, where "Up" indicates significant up-regulation (*p*-value < 0.05 and log2FoldChange > 0), and "No diff" indicates no significant difference.

## 5 Conclusion and outlook

The findings of the present study demonstrated that treatment with an appropriate dose of resveratrol (50 mg/kg) elevated testosterone levels in male Arctic foxes, which was consistent with the upregulated expression of key genes involved in the testosterone biosynthetic pathway, including *HSD3B* and *CYP11A1*. The hypothesis regarding negative feedback regulation under high-dose resveratrol exposure, proposed based on correlation analysis, warrants further validation using functional experiments such as estradiol measurement, receptor blockade assays, detection of pituitary LH expression, or metabolic flux tracing. Furthermore, systematic and in-depth investigations are still needed to identify the optimal dose window that effectively enhances net testosterone production, while avoiding excessive activation of the cortisol synthesis pathway linked to Cushing’s syndrome.

## Ethics statement

The experimental protocol was approved by the College of Animal Science and Technology, Jilin Agricultural University, and its Ethics Committee (20241022).

## Declaration of generative AI and AI-assisted technologies in the writing process

During the preparation of this manuscript, the authors used DeepSeek for English translation and language polishing.

## CRediT authorship contribution statement

**MA Limin:** Writing – original draft, Methodology, Formal analysis, Conceptualization. **Duan Fuwen:** Validation, Investigation, Data curation. **Liu Zijian:** Validation, Methodology, Investigation. **Tian Yu:** Validation, Investigation, Formal analysis. **Wu Min:** Supervision, Resources, Project administration. **Wei Gongqing:** Writing – review & editing, Supervision, Project administration, Funding acquisition.

## Declaration of competing interest

The authors declare no conflict of interest.

## Data Availability

The datasets generated during and/or analyzed during the current study are available from the corresponding author on reasonable request.
